# Physicochemical Exploration and Computational Analysis of Bone After Subchronic Exposure to Kalach 360 SL in Female *Wistar* Rats

**DOI:** 10.3390/toxics13060456

**Published:** 2025-05-29

**Authors:** Latifa Hamdaoui, Hafedh El Feki, Marwa Ben Amor, Hassane Oudadesse, Riadh Badraoui, Naila Khalil, Faten Brahmi, Saoussen Jilani, Bandar Aloufi, Ibtissem Ben Amara, Tarek Rebai

**Affiliations:** 1 Laboratory of Induced Diseases and Development, Sfax Faculty of Medicine, University of Sfax, Sfax 3029, Tunisia; benamor.ayadi.marwa@gmail.com (M.B.A.); tarekrebaifmsf@gmail.com (T.R.); 2 Faculty of Sciences of Sfax, University of Sfax, Sfax 3029, Tunisia; hafed.elfeki@yahoo.fr; 3 University of Rennes, CNRS, ISCR-UMR 6226, F-35000 Rennes, France; hassane.oudadesse@univ-rennes1.fr; 4 Laboratory of General Biology, Department of Biology, College of Science, University of Ha’il, Ha’il 81451, Saudi Arabia; riadh.badraoui@fmt.utm.tn (R.B.); f.brahmi@uoh.edu.sa (F.B.); s.jilani@uoh.edu.sa (S.J.); b.alofi@uoh.edu.sa (B.A.); 5 Section of Histology-Cytology & Cytogenetics, Faculty of Medicine of Tunis, University of Tunis El Manar, La Rabta-Tunis 1007, Tunisia; 6 Department of Population and Public Health Sciences, Boonshoft School of Medicine, Wright State University, Dayton, OH 45435, USA; naila.khalil@wright.edu; 7 Laboratory of Medicinal and Environment Chemistry, Higher Institute of Biotechnology, University of Sfax, Sfax 3000, Tunisia; ibtissem.benamara@isbs.usf.tn; 8 Tropical Biome and Immunopathology CNRS UMR-9017, Inserm U 1019, University of Guiana, 97300 Cayenne, France

**Keywords:** Kalach, hydroxyapatite, FTIR, XDR, osteocalcin, in silico modeling

## Abstract

Glyphosate (N-phosphonomethylglycine) is a widely used organophosphorus herbicide that inhibits the shikimate pathway, a crucial metabolic route responsible for the synthesis of aromatic amino acids in plants and certain microorganisms. Due to its broad-spectrum activity, glyphosate serves as the main active ingredient in various commercial herbicide formulations, including Roundup and Kalach 360 SL (KL). It poses a health hazard to animals and humans due to its persistence in soil, water erosion, and crops. The aim of our study was to continue the previous research to explore the impact of KL on bone using physico-chemical parameters and in silico studies after exposing female *wistar* rats for 60 days. The in silico study concerned the assessment of binding affinity and molecular interactions using computational modeling approach. The rats were allocated into three experimental groups: group 1 (*n* = 6) served as controls, while groups 2 and 3 received low and high doses (Dose 1: 126 mg/Kg and Dose 2: 315 mg/Kg) of KL dissolved in water, respectively. All rats were sacrificed after 60 days of exposure. XRD and FTIR spectrum analysis of bone tissues in female rats showed significant histoarchitectural changes associated with bone mineralization disruption. Our results have demonstrated that sub-chronic exposure of adult female rats to KL causes bone rarefaction, as confirmed by a previous histological study. This physico-chemical study has further confirmed the harmful impact of KL on the crystalline fraction of bone tissue, composed of hydroxyapatite crystals. In addition, the computational analyses showed that glyphosate binds to 3 Glu form of osteocalcin (3 Glu-OCN) (4MZZ) and decarboxylated osteocalcin (8I75) with good affinities and strong molecular interactions, which justified and supported the in vivo findings. In conclusion, KL may interfere with hydroxyapatite and osteocalcin and, therefore, impair bone remodeling and metabolism.

## 1. Introduction

Kalach (KL) is a widely used organophosphorus herbicide in Tunisia, containing 41.5% glyphosate in the form of N-(phosphonomethyl)glycine as its active ingredient [1,2]. Moreover, this herbicide contains 15.5% surfactant and 43% water. The first glyphosate-based herbicide (GBH) is RoundUp, and subsequent formulations such as Glyphogan, Touchdow, or KL, are blends of GLP and various adjuvants designed to enhance its penetration in plants and increase effectiveness. Many of these adjuvants are surfactants, including polyethoxylated tallow amine (POEA) [3].

Humans throughout the world are exposed daily to low levels of environmental contaminants induced by pesticide residues [4,5,6], such as GBH. It has been reported that pesticides alter the remodeling process of bones and the development of the skeletal system by their hormonal regulation. Moreover, pesticides have been shown to induce skeletal abnormalities and malformations in both experimental animal models and wildlife species [7,8]. 

Bone formation in the mammalian skeleton is primarily mediated by a coordinated network of specialized cells. Osteoprogenitor cells are essential for sustaining the osteoblast population and preserving bone mass. Osteoblasts actively synthesize the bone matrix at sites of bone formation, whereas osteocytes, which are embedded within the mineralized matrix, contribute to structural integrity and serve as key regulators of bone remodeling. In addition, quiescent bone-lining cells provide a protective layer on bone surfaces. The remodeling process is tightly regulated and involves a sequential interplay between osteoclast-mediated bone resorption and osteoblast-driven bone formation, with osteocytes playing a pivotal regulatory role in this dynamic cycle [9,10,11]. 

The composition of bone underlies its distinctive mechanical strength, protective capacity, and homeostatic functions. Although this composition can vary depending on factors such as age, anatomical location, diet, and overall health, adult mammalian bone typically consists of 50–70% mineral content, 20–40% organic matrix, 5–10% water, and approximately 3% lipids. The development, maintenance, and remodeling of the skeletal system in humans and animals are primarily governed by three major cell lineages: the osteogenic lineage, which gives rise to osteoblasts and osteocytes responsible for bone formation and structural integrity; the osteoclastic lineage, derived from hematopoietic precursors, which mediates bone resorption; and the chondrogenic lineage, which produces chondrocytes essential for cartilage formation and endochondral ossification [12]. Osteoblasts secrete hydroxyapatite crystals (HPA) [Ca_10_(PO_4_)_6_(OH)_2_] and calcium to increase mineralization to produce a new set of cells called osteocytes; osteocytes are mature bone cells, and osteoclasts are hematopoietic in origin [12].

It has been reported that GBH can contribute to osteoporosis through its potential effects on bone metabolism [13]. Osteoporosis is a bone disease marked by reduced bone density and structural degradation of bone tissue, which results in greater susceptibility to fractures and increased bone fragility. The condition primarily results from an imbalance between osteoblast and osteoclast activity, where bone resorption by osteoclasts exceeds bone formation by osteoblasts [14]. Bone remodeling is a dynamic process influenced by several biological, mechanical, hormonal, and environmental factors. Of these factors, we found that osteocalcin, a non-collagenous protein found in bone, plays a key role in promoting bone formation, regulating glucose metabolism, supporting testosterone production, and preserving muscle mass. Osteocalcin does not function as a hormone in bone tissue, it is essential for the alignment of apatite crystals [15]. Perturbation of these factors has the potential to disrupt the normal mineralization process, leading to an abnormal or pathological composition of bone [16].

X-ray diffraction (XRD) and Fourier Transform Infrared Spectroscopy (FTIR) are widely utilized techniques for investigating bone disorders, particularly those involving alterations in bone composition and mineralization [17].

This study is a continuation of previous work in our laboratory [7], which suggested that KL induces bone rarefaction in female Wistar rats. By building on that previous work in the present study, the objective is to confirm that KL causes osteoporosis by physico-chemical exploration and in silico study.

We investigated the effects of KL on bone mineralization and composition using FTIR, XRD, bone study microanalysis-energy spectrometry (EDX), and histological exploration to assess potential changes in mineral features associated with osteoporosis induced by GBH. The binding affinity and the molecular interactions, of glyphosate with the 3 Glu form of osteocalcin (3 Glu-OCN) (4MZZ) and decarboxylated osteocalcin (8I75) were also assessed using computational assays.

## 2. Materials and Methods

### 2.1. Chemicals Used in Our Study

**Figure 1:**
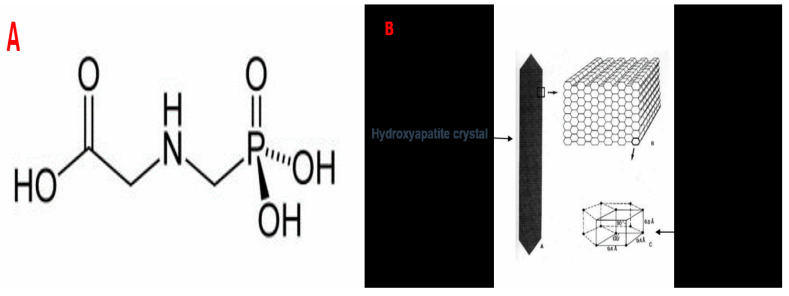
(**A**) Represents the molecular structure of GLP. (**B**) Represents the mineral substance of bone tissue, which is a calcium phosphate crystallized in the form of hydroxyapatite.

The herbicide applied in this study was a commercial formulation of Kalach (KL), comprising 360 g/L of glyphosate (GLP) as the active ingredient, classified as an organophosphorus compound (Figure 1). The GLP, isopropylamine salt of n phosphonomethylglycine represented 41.5% of KL. Indeed, this herbicide also contains 15.5% of surfactant and 43% of water.

### 2.2. Animals

A total of eighteen adult female Wistar rats, each weighing approximately 200–220 g, were obtained from the Central Pharmacy (SIPHAT, Ben Arous, Tunisia) for the purpose of assessing the impact of sub-chronic herbicide exposure on bone development. The animals were housed under standardized laboratory conditions, including a constant temperature of 22 °C, relative humidity of 60%, and a 12 h light/dark cycle.

The Ethics Committee in Research approved the experimental protocol and all efforts were made to minimize animal suffering and reduce the number of animals used.

### 2.3. Experimental Design

Our protocol was the same as that in our previous study [7]. Rats were divided into 3 groups (*n* = 6) as follows:

Group 1: control (did not receive any substances and received water only by gavage);

Group 2 was administered 0.07 mL of KL, diluted in 1 mL of water, corresponding to a dose of 126 mg G/Kg (Dose 1).

Group 3 received 0.175 mL of KL, also diluted in 1 mL of water, delivering a dose of 315 mg G/Kg (Dose 2).

The treatment period was 60 days and the KL solution was given by oral administration. Multiple studies have explored GLP’s toxicity thresholds. For instance, in humans, a no-observed adverse effect level (NOAEL) of 50 mg/kg of body weight per day was established in 2015 [18]. In rats, the long-term toxicity threshold was determined as 350 mg/kg/body weight/day, with an LD_50_ of 5 g/kg/body weight/day [19]. These studies have reported a wide range of administered GLP doses, spanning from 40 to 50,000 mg/kg of body weight [20]. In our study, the determined LD_50_ and the dose administered to rats are lower than the lethality and toxicity thresholds, respectively, and are consistent with the literature.

The LD_50_ of KL was determined and documented in the literature and then confirmed through testing in our laboratory [7]. The present study is a continuation of our previous study, where we chose two doses: Dose 1 contains 126 mg of GLP/Kg and Dose 2 contains 315 mg of GLP/Kg. These doses are within the long-term toxicity threshold of 350 mg/kg/body weight/day in rats [18].

All experimental procedures, including animal handling, sampling, and euthanasia, were conducted in compliance with the guidelines of the National Institutes of Health for the care and use of laboratory animals, and were approved by our institution’s Regional Ethics Committee for Animal Experimentation (CEREAS). The protocol code is CEREAS-FJ4625/4025.

### 2.4. Euthanasia

The *Wistar* rats were humanely euthanized under anesthesia through an intraperitoneal injection of midazolan and ketamine, as previously published [1,7]. The right distal femurs were carefully dissected, and their lengths were recorded. All experimental procedures were carried out in accordance with the guidelines approved by the local Animal Ethics Committee. Subsequently, the bones from each group were pulverized into a fine powder and subjected to physico-chemical analyses for characterization.

### 2.5. Infrared Spectroscopic (FTIR) Analysis of Bone Sample

The FTIR technique was employed to identify the chemical composition of KL and the bonds between atoms. Bone samples were ground using a liquid nitrogen-cooled colloid mill (Retsch MM 200, Retsch, Haan, Germany) and subsequently subjected to vacuum freeze-drying (MAXI dry lyo, Heto, Cabaana, MI, USA). Pellets for FTIR analysis were prepared by combining the ground bone with KBr at a ratio of 1 mg sample to 100 mg KBr under vacuum conditions. The equipment utilized for FTIR analysis was the Nicolet Magna-IR 550 spectrometer manufactured in Madison, WI, USA [21]. FTIR spectra were collected using a Bomem MB157 spectrometer (Québec, QC, Canada) operating in the 4000–400 cm^−1^ range and purged with dry air. Each spectrum was obtained by averaging 400 interferograms with a resolution of 4 cm^−1^.

### 2.6. X-Ray Diffraction (XRD) Analysis

The XRD pattern of powder samples was measured on a D8 Bruker diffractometer (Billerica, MA, USA) controlled by an IBM PC microcomputer (Armonk, NY, USA). It is used to analyze the structure of bone samples. The diffract meter operated at 40 kV and 30 mA at over a 2θ range of 10–70 [22].

### 2.7. Optical Emission Spectrometry (ICP-OES)

Optical Emission Spectrometry, or ICP-OES (Inductively Coupled Plasma–Optical Emission Spectrometry), is a physical method of chemical analysis that allows the simultaneous quantification of almost all elements. It is a quantitative method with a sensitivity lower than μg/g or ppm [23]. This technique is capable of measuring the concentrations of certain elements such as calcium, phosphorus, sodium, strontium, etc [22].

### 2.8. Computational Study

The crystal structure of the 3 Glu form of osteocalcin (3 Glu-OCN) (pdb id: 4MZZ) and decarboxylated osteocalcin (DOC) (pdb id: 8I75) has been retrieved from the RCSB database. Their active sites have been targeted to assess the molecular interactions. The three-dimensional chemical structure of GLP has been collected from the PubChem website. GLP, along with 3 Glu-OCN and DOC, has been subjected to CHARMm force field, as previously described [24,25,26,27], after removing water molecules and adding both Kollman charges and polar hydrogens. Bond categories and binding scores were analyzed, as previously assessed [28,29,30]. The major reason behind targeting 3 Glu-OCN and DOC is their key role in bone physiological and pathophysiological remodeling.

### 2.9. Statistical Analyses

Statistical analysis was carried out using SPSS software (version 26, SPSS Inc., Chicago, IL, USA). Parametric quantitative data were presented as mean ± standard deviation (SD) and analyzed using one-way ANOVA followed by a post hoc test with a Duncan test. A *p*-value of less than 0.05 was considered statistically significant.

## 3. Results

### 3.1. FTIR Results

**Figure 2:**
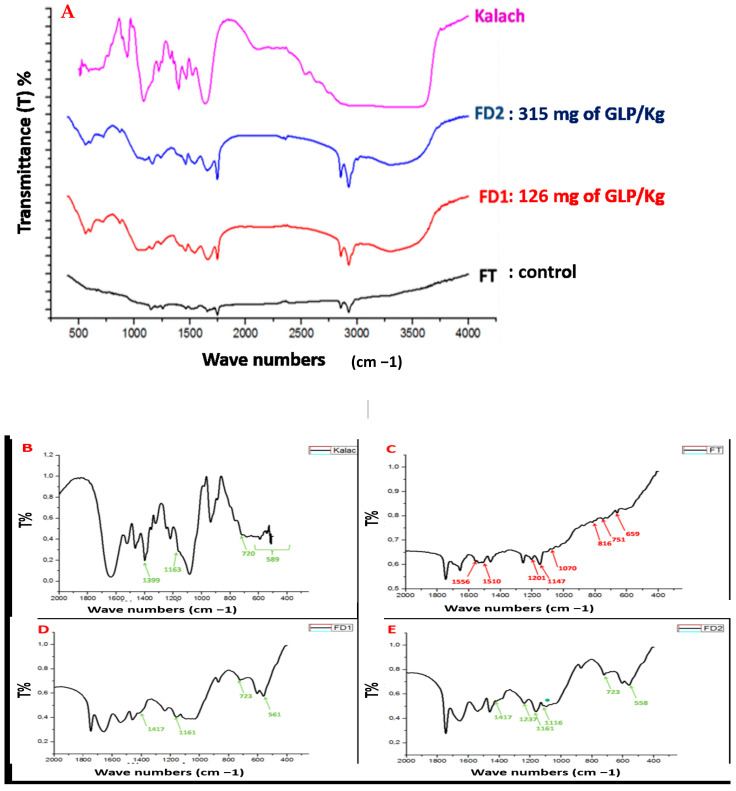

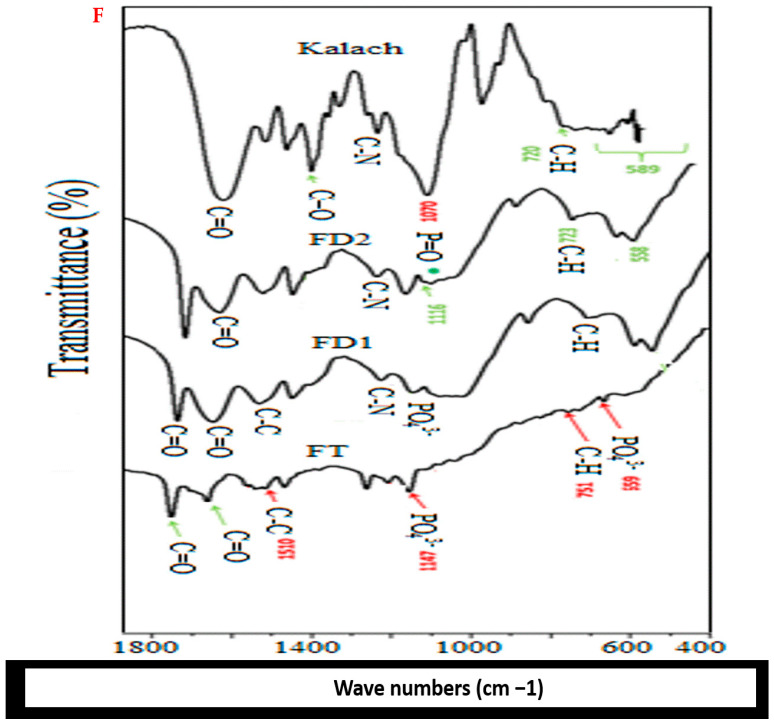
FTIR spectra of control and treated rats. (**A**) displays the FTIR spectra of powdered samples obtained from the femurs of control rats, KL-treated rats at varying doses, and the KL solution itself. (**B**–**E**): Spectra of the FTIR impact of KL administered at two different doses in rats treated for 60 days on bone samples. we selected the range between 250 and 2000 cm^−1^, which clearly indicates significant modifications in the treated groups. 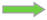 The newly appeared bands; 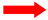 The bands disappeared compared to the control group. FT: Female rats of the control group; FD1: Female rats treated with Dose 1 of KL; FD2: Female rats treated with Dose 2 of KL. The green asterisk represent the newly appeared bands. (**F**): FTIR analysis allowed us to detect groups specific to KL in the bones of treated rats.

Figure 2A displays the FTIR spectra of powdered samples obtained from the femurs of control rats, KL-treated rats at varying doses, and the KL solution itself. In order to better observe the influence of KL on bone tissue through the FTIR technique, we selected the range between 250 and 2000 cm^−1^, which clearly indicates significant modifications in the treated groups, as shown in Figure 2B–E.

Our findings suggest that KL induces bone disturbances when administered through gavage for 60 days. These figures (Figure 2B–E) demonstrate the presence of common bands between the spectra of bones from control rats and the spectra of rats treated with KL doses 1 and 2.

The spectra collectively exhibit characteristic bands associated with the vibrational modes of the phosphate group (PO_4_^3−^), detected at 546, 602, 659, 1147, 1201, and 1253 cm^−1^.

In the KL-treated groups, several spectral shifts were observed, including the disappearance of some bands and the emergence of new ones, along with changes in wavenumber values compared to the control group (Table 1).

The carbonate (CO_3_^2−^) bands identified at 816, 1510, 1536, 1556, and 1563 cm^−1^ exhibited slight shifts following KL exposure, as detailed in Table 1. These alterations indicate that KL treatment induced structural modifications in bone composition, reflected by the appearance and disappearance of specific vibrational bands.

FTIR analysis allowed us to detect groups specific to KL in the bones of treated rats (Figure 2F). The bands related to these groups appear on the FTIR spectrum around the following:

The 1637 cm^−1^ band corresponding to the C=O bond;
The 1400 cm^−1^ band corresponding to the C-O bond;
The 1218 cm^−1^ band corresponding to the C-N bond;
The 720 cm^−1^ band corresponding to the C-H bond

On the other hand, the bands of the C=O, C-O, and C-H groups in rats treated with KL (FD1 and FD2) are more intense compared to the control group. Considering that GLP is a molecule characterized by the presence of two acidic functions, a carboxylic acid group (-COOH) and a phosphonic group, as well as an amine function (Figure 1A), this is evidenced in our FTIR results by the presence of the C-N group. This latter group, detected in the FTIR spectrum of the bones of treated rats, constitutes the most toxic element of KL.

### 3.2. XRD Results

**Figure 3:**
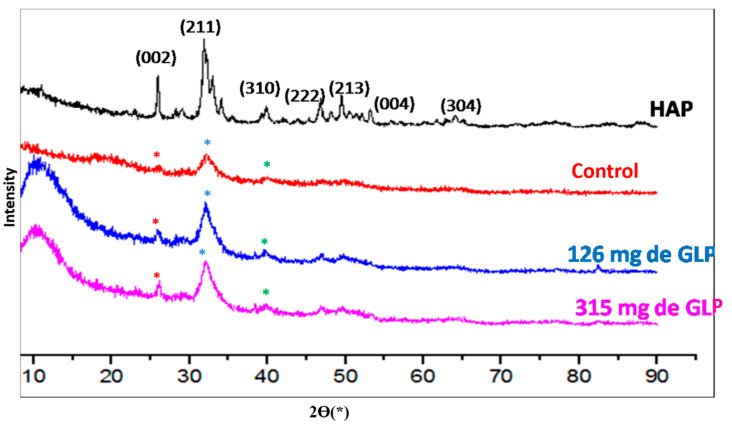
X-ray diffractogram findings of control and treated rats. Black line: X-ray diffractogram spectra of HAP. Red line: X-ray diffractogram spectra of control group. Blue line: X-ray diffractogram spectra of rats treated by 126 mg of GLP. Purple line: X-ray diffractogram spectra of rats treated by 315 mg of GLP.

The diffraction patterns of HAP and control femur samples were used as references to assess the impact of KL herbicide at varying doses (Table 2). After 60 days of exposure to KL, the XRD revealed characteristic peaks of HAP crystals at approximately 26, 32, 40, 46.7, 50, 53, and 64° (2θ), corresponding to the (002), (211), (310), (222), (213), (004), and (304) planes of HAP crystals, respectively (Figure 3).

Table 2 indicates shifts in the peak positions, particularly for the (002), (211), and (310) planes. These shifts suggest that the presence of glyphosate (G) leads to a reduction in the 2θ values of these peaks. A decrease in 2θ is associated with an expansion of the crystallographic lattice parameters (a = b, c), indicating that KL exposure contributes to an enlargement of the crystal unit cell in bone tissue. These structural modifications align with the FTIR results, which also revealed perturbations in the mineral phase of bone due to herbicide incorporation.

### 3.3. Bone Microanalysis: Energy-Dispersive X-Ray Spectroscopy (EDX) and ICP Results

**Figure 4:**
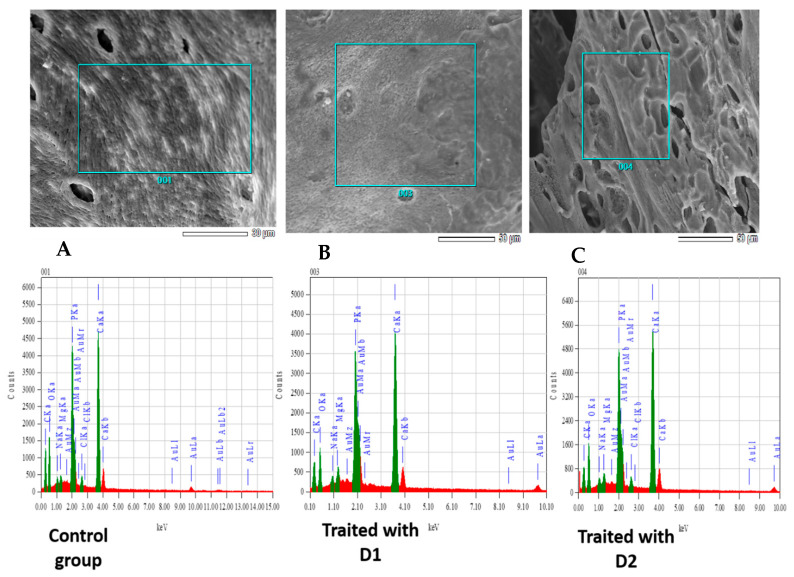
The EDX spectra and elemental distribution maps illustrate the femoral composition of control rats and those treated with KL at doses D1 and D2. (**A**) EDX spectra of control group. (**B**) EDX spectra of rats treated by 126 mg of GLP. (**C**) EDX spectra of rats treated by 315 mg of GLP.

Elemental distribution within bone samples was assessed using energy-dispersive X-ray spectroscopy (EDX) in conjunction with X-ray backscattering imaging. Representative elemental maps for the control group and the groups treated with 126 mg/kg and 315 mg/kg of glyphosate (G) are presented in Figure 4A–C, respectively. The calcium-to-phosphorus (Ca/P) ratio, indicative of bone mineral composition, was found to be 2.58 in the control group. In contrast, groups exposed to Dose 1 (126 mg/kg) and Dose 2 (315 mg/kg) exhibited elevated Ca/P ratios of 2.79 and 2.67, respectively (Table 3).

### 3.4. Computational Results and Interaction Assays

**Figure 5:**
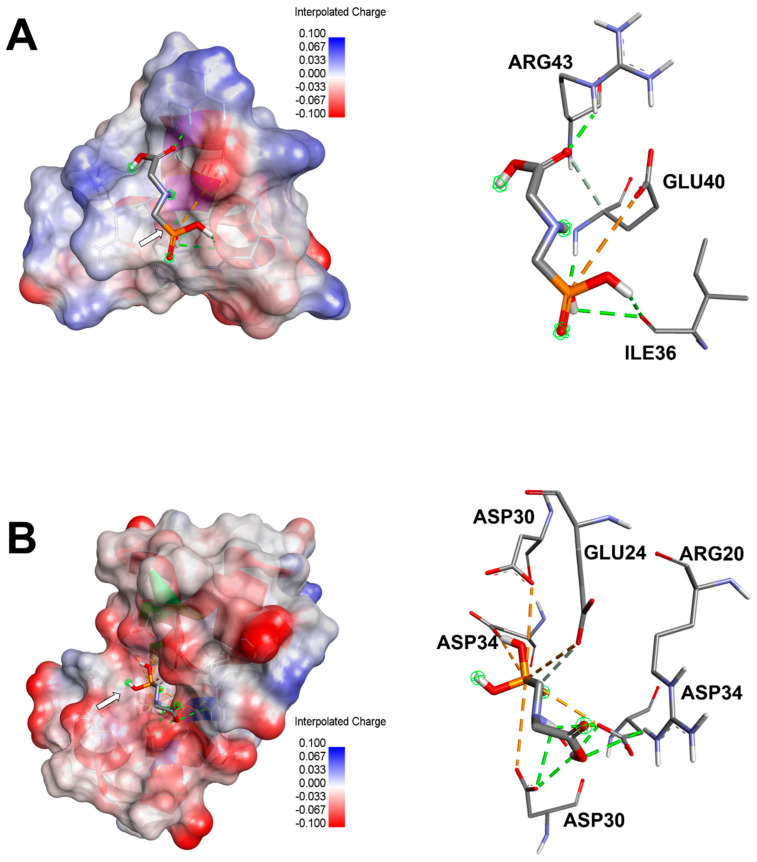
Three-dimensional illustrations of macromolecule–ligand complexes (left) and their corresponding 3D diagrams of interactions (right) for the glyphosate complexed with the (**A**) 3 Glu form of osteocalcin (3 Glu-OCN) (4MZZ) and (**B**) decarboxylated osteocalcin (8I75).

**Figure 6:**
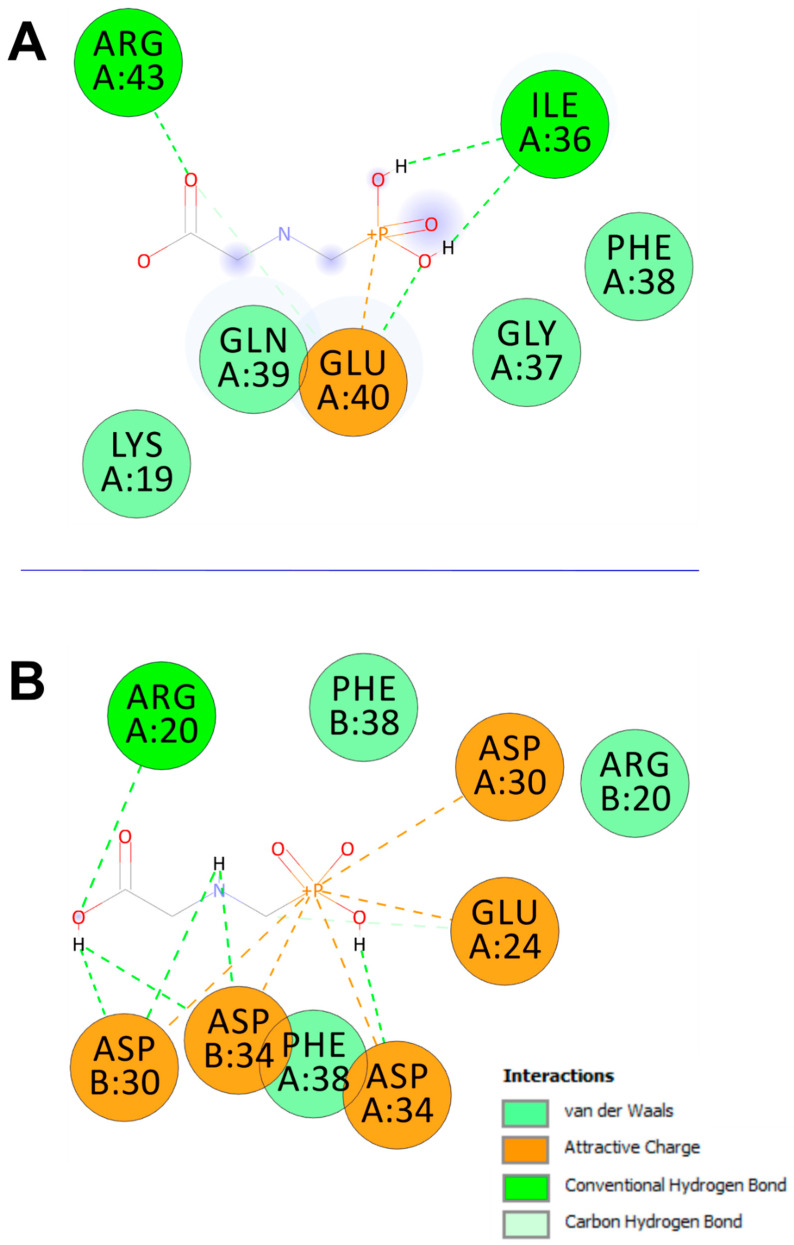
Two-dimensional diagrams of interactions of the glyphosate complexed with (**A**) 3 Glu form of osteocalcin (3 Glu-OCN) (4MZZ) and (**B**) decarboxylated osteocalcin (8I75).

Glyphosate was predicted to bind 3 Glu-OCN and DOC with acceptable affinities and root mean square deviation (RMSD). The binding affinities were not strong (lower than −6 kcal/mol) but all negative and reached −3.5 and −4.2 kcal/mol, for 3 Glu-OCN and DOC, respectively (Table 4). GLP was further analyzed for bond category, molecular interactions, and tight embedding with the targeted receptors. Our results exhibited that GLP established acceptable molecular interactions that included conventional H-bonds associated with a network of attractive charges and carbon H-bonds. The established interactions concerned several key residues and tight embedding (<2.5 Å). The predicted closest interactions of GLP concerned GLU40 (three times) and twice for each of ARG43 and ILE36 of the 3 Glu-OCN macromolecules. It concerned ASP34 (5 times), ASP30 (4 times), GLU24 (twice), and once for ARG20 within DOC macromolecule. These computational results paralleled the in vivo findings (Figure 5 and Figure 6).

## 4. Discussion

To investigate the impact of KL on bone remodeling, we employed an in silico approach in combination with analytical techniques, including Inductively Coupled Plasma (ICP) analysis for calcium and phosphorus content, as well as FTIR, XRD, and EDX for physico-chemical characterization. This study is a continuation of a previous study and investigates the direct effect of KL on the HPA of bone using physico-chemical explorations. To our knowledge, our paper represents the first report on the exploration of the interaction of GLP in the bone tissue of female *Wistar* rats. Earlier research has indicated that bone remodeling may be adversely affected by exposure to xenobiotic compounds, potentially leading to various toxicological effects [7,30,31,32]. The teratogenic effects of GBHs have been documented in various species, including fish, rats, mice, and chickens, where bone and brain malformations have been noted [33,34,35].

In order to confirm these observations, it is essential to conduct FTIR analyses on the bone samples from rats treated with KL and compare them to the control group. The disturbance in bone tissue is indicated by the following four points in the case of an infrared exploration, such as broadening of bands, shifting of bands, disappearance of bands, and the appearance of new bands [17,36,37]. Therefore, our FTIR analyses clearly show shifts in the wave number values of the OH^−^, PO_4_^3−^, and CO_3_^2−^ groups in the groups treated with KL compared to the control group. This further confirms the disruptive effect of KL for both doses, D1 and D2, on bone remodeling through the interaction of this molecule with bone molecules. These observations suggest significant changes in the bone spectra of rats treated with KL, as compared to the control group.

We noted that there is a disappearance of four bands corresponding to the vibrations of the CO_3_^2−^ group observed around 1556, 1510, 816, and 751 cm^−1^ in both groups treated with 1/10 and 1/4 of the LD_50_ of KL. Moreover, we found a disappearance of two bands corresponding to the vibrations of the PO_4_^3−^ group observed around 1147 and 1070 cm^−1^ in both groups treated with KL, D1, and D2 (FD1 and FD2). Indeed, our results showed a disappearance of a band corresponding to the vibration mode of the OH- group (non-shifted) observed around 659 cm^−1^. Compared to the bone of the control group (FT), there is an appearance of new bands in the spectra of treated rats: a band corresponding to the vibration mode of CO_3_^2−^ observed around 1417 cm^−1^ in both groups treated with KL, D1, and D2 (FD1 and FD2). These observations suggest significant changes in the bone spectra of rats treated with KL, including the disappearance of bands and the appearance of new bands compared to the control group.

We observe the appearance of two new bands corresponding to the vibration modes of the PO_4_^3−^ group observed around 1160 and 1116 cm^−1^ in both groups treated with KL, D1, and D2. Previous studies of our group examined the toxic impact of imidacloprid (IMI) on bone health, particularly its role in inducing osteoporosis in female rats, and evaluated the potential protective properties of *Urtica urens* L. Our findings indicated that IMI exposure led to osteoporosis, as evidenced by reduced levels of calcium (Ca) and phosphorus (P). XRD analysis revealed peak shifts in IMI-treated rats compared to controls, and the FTIR method demonstrated notable band shifts associated with IMI exposure [37].

As mentioned, GLP consists of a carboxylic acid group (-COOH). The effect of this acid is well known for demineralizing hard tissues by forming more soluble calcium salts, as highlighted by Feitosa et al. [38]. Indeed, an acidic proton should participate in the release and dissolution of calcium from the mineral phase of the bone, leading to an increase in the proportion of demineralized collagen. This effect is explained by the condensation of a carboxylic acid group (-COOH). Furthermore, we believe that all acid monomers react with the mineral phase of the bone (HPA).

Although there are some differences between the diagram of XRD of the control bone and that of the treated rats, we attempted to draw relevant conclusions based on these diagrams, and the results are summarized in Table 3. According to these results, we observed shifts in the values of Ө. Indeed, the presence of KL at different doses (D1 and D2) decreases the values of Ө for the peaks at 26°, 32° and 40° (2θ). As Ө decreases, the crystallographic parameters (a = b, c) increase, leading to an enlargement of the bone lattice volume [37]. This result is in good agreement with that obtained by FTIR, which showed a disturbance in the bone caused by the introduction of the herbicide into the crystalline mineral phase of the bone. These results indicate that the crystallization evolution of the HPA layer in rats treated with D1 and D2 of KL is disturbed when compared with the control group.

Furthermore, a study conducted by Jebahi et al. [22] demonstrated, using XRD diagrams, that the glass–chitosan composite (46S6-CH17) implanted at the femoral condyle of ovariectomized rats exhibits a new peak at 29° (2θ) compared to pure glass (pure bioactive glasses 46S6). They also showed that the shift in the center of the glass diffraction halo and the appearance of new peaks highlight interactions between the bioactive glass and the chitosan polymer. There is an association between the orthorhombic system of chitosan and the amorphous structure of the glass to form a new structure of glass–chitosan composites [22]. After 15 days of implantation, the XRD pattern of the 46S6-CH17 implant at the femoral condyle site exhibits two peaks at 26 and 32° (2θ), characteristic of the HPA phase. These peaks correspond, respectively, to the reflections of the (002) and (211) planes of HPA. This result confirms the formation of the HPA layer.

In the present study, sub-chronic exposure to KL in rats leads to a significant decrease in bone content of calcium and phosphorus. The phospho/calcic ratio obtained here is 2.58 for the control group, while for the groups treated with D1 and D2, the ratios are 2.79 and 2.67, respectively (Table 3). These results confirm our histological findings and the observed osteoporotic aspects in the previous study [7]. These results allow us to propose a hypothesis: the chemical composition of KL, specifically GLP, contains acids known for their destructive effects on bone. Raynaud et al. reported that variations in the ratio Ca/P are associated with changes in both the structure and texture of the analyzed powder. Mackay highlighted that the presence of hydrogen phosphate (HPO_4_^2−^) accounts for a lower Ca/P ratio compared to the stoichiometric value of 1.67, contributing to structural disruption within the apatite matrix [39,40].

Moreover, our ICP results are consistent with previous findings from our group, indicating that KL exposure can interfere with various metabolic pathways—particularly those involving Ca and P—and adversely affect organs such as the thyroid, kidneys, liver, and ovaries, all of which play key roles in bone metabolism.

In our research group, we focused on the effect of acute and sub-chronic exposition to pesticides such as KL in female and male rats. Our previous findings showed a decline in body weight and body weight gain [1,7]. This is consistent with previous research involving mice exposed to Roundup^®^ for acute period [41]. Weight loss serves as a critical indicator of toxicity and reflects the capacity of GBHs to stimulate ROS production [1,7]. Therefore, it can be inferred that KL induced systemic toxicity in treated rats. In our previous findings, we noted a decrease in bone weights, when compared with controls. These results can be associated with high or low rates of remodeling with the imbalance between resorption and formation [7].

**Figure 7:**
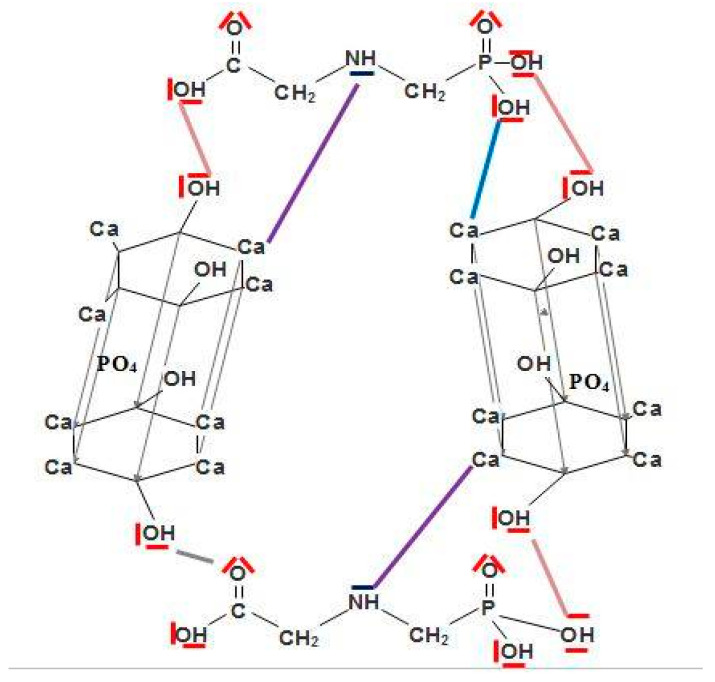
Reaction mechanism and interaction of GLP with bone. Purple line: represent interaction between Ca of HAP and NH of GLP. Blue line: represent interaction between Ca of HAP and OH of GLP. Red line represent interaction between oh of HAP and OH of GLP.

The active ingredient in KL is GLP, which contains an organic acid group that can disturb bone structure by attacking its lattice (Figure 3) through interactions with the active sites of HPA. As shown in Figure 3, there is an interaction between the groups of our herbicide compound (GLP) and bone, following three possibilities. Indeed, the nitrogen atom (N) of the GLP molecule can easily bind to the hydrogen atom located on the helical senary axis (A6) of HPA. Moreover, the oxygen atom of GLP can easily form a chemical bond with Ca^2+^ in the mineral part of the bone. Finally, the phosphorus atom of GLP can also interact either with the oxygen atom of the phosphate group located in the skeleton of the structure or with that of the hydroxyl group located in the tunnel of HPA. Hatched lines in Figure 7 mark the reaction mechanisms, using the developed formulas of bone (HPA) and GLP.

Glyphosate was predicted to bind 3 Glu-OCN and DOC with acceptable affinities that reached −3.5 and −4.2 kcal/mol, respectively (Table 4). These negative binding affinities for the two-targeted receptors supported their potential effects. It has been reported that variation in binding affinities depended mainly on the tridimensional chemical structure and geometry of the studied compounds [25,27,29]. The binding affinity was predicted to be better for DOC. GLP was further analyzed for bond category, molecular interactions, and tight embedding with the targeted receptors. Our results exhibited that GLP established acceptable molecular interactions that support its potential toxicological outcomes. The established interactions included conventional H-bonds associated with a network of attractive charge and carbon H-bonds. Previously, it has been reported that a rich network of bonds, particularly attractive charges and H-bonds, enhanced the stability of the ligand–receptor complex [25,26,42]. Furthermore, the established interactions concerned several key residues and deep embedding (<2.5 Å), which have been commonly reported to be associated with several toxicological effects [43,44,45]. The closest interactions of GLP concerned GLU40 (three times, and occurred twice for both ARG43 and ILE36 of the 3 Glu-OCN macromolecule. It concerned ASP34 (five times), ASP30 (four times), GLU24 (twice), and once for ARG20 within the DOC macromolecule (Figure 5 and Figure 6). Taken together, the computational findings showed that the impact of GLP on bone remodeling and composition is thermodynamically possible. This has already been reported in the in vivo approaches of the current and previous studies in rats. These findings support the negative impacts of toxicants, particularly pesticides, such as GLP [7,44,45,46].

Evidence indicates that commercial GBH formulations exhibit higher toxicity compared to GLP alone. This enhanced toxicity is primarily due to the inclusion of co-formulates such as surfactants, adjuvants, and diluents, which facilitate the systemic uptake and translocation of GLP in plants. These co-formulates, particularly surfactants, may exert additive or synergistic effects on endocrine disruptive pathways and can affect bone tissue indirectly [41]. Consequently, the toxicological profile of GBHs is complex, involving multiple interacting components and mechanisms [47]. Limited research has been conducted on the toxic effects of GBHs on bone tissue, and the present study constitutes an initial contribution to addressing this issue.

In the same context, we can cite a recent study by McGehee that investigated the impact of GLP on human osteoblast cell activity. The study demonstrated that prolonged exposure to GLP led to a dose-dependent increase in osteoblast proliferation, with the most significant effect observed at the lower concentration of 0.007 mg/mL. Furthermore, a marked enhancement in mineralization was detected at both 0.0007 and 0.007 mg/mL GLP concentrations [48].

Finally, this study provides the first detailed exploration of KL’s impact on bone tissue by physico-chemical and in silico analysis, revealing its toxicity. These findings underscore the need for further research to better understand the long-term implications of KL, particularly regarding its potential impact on human bone health.

## 5. Conclusions

Our study indicated that the toxicity of KL and GBH has affected the bone mineralization of female rats. It confirmed that KL-induced osteoporosis is caused by the interaction of the active ingredient GLP, which contains an organic acid group, with the active sites of the ICP method and physico-chemical techniques (FTIR, XRD, and EDX), confirming the harmful impact of KL on the crystalline fraction of bone tissue. Therefore, KL can disturb bone structure directly and indirectly through its interaction with hydroxyapatite and osteoclacin, respectively, as outlined by the in vivo and computational results.

## Figures and Tables

**Table 1:** Displacements, Desperations and Apparition of new bands for the different groups of rats.

Wave Numbers (cm^−1^)/Group	CO_3_^2−^						PO_4_^3−^								OH- Displaced	
Control	1563	1556	1536	1510	-	816	1253	1201	-	1147	-	602	659	546	718	751
Group 2 (126 mg of GLP/Kg)	-	-	1543	-	1417	-	1240	-	1161	-	-	605	-	561	723	-
Group 3 (315 mg of GLP/Kg)	-	-	1538	-	1417	-	1237	-	1160	-	1116	605	-	558	720	-
Kalach	-	-	1523	-	1399	767	1247	1219	1163	-	-	589	720		1523	687

**Table 2:** DRX in different groups of rats.

Group/Peak	DRX		
	(002)	(211)	(310)
**HPA**	26	33	41
**Control**	26	33	41
**Group 2** **(126 mg of GLP/Kg)**	25.97	32.16	39.81
**Group 3** **(315 mg of GLP/Kg)**	25.12	32.33	39.65

**Table 3:** Bone and KL levels of calcium and phosphorus in control and treated rats.

	Ca mg/g of Tissu	P mg/g of Tissu	Ca/P
Control	35.91 ± 0.07	13.80 ± 0.18	2.60 ± 0.03
Group 2 (126 mg of GLP/Kg)	37.10 ± 1.16	13.27 ± 0.19 **	2.79 ± 0.11 *
Group 3 (315 mg of GLP/Kg)	33.86 ± 1.20 *	12.66 ± 0.41 **	2.67 ± 0.13 *
	**Ca ppm**	**P ppm**	**Ca/P**
Kalach	11.96 ± 1.28	31.66 ± 22.36	0.78 ± 0.87

Data are presented as mean ± SD. Statistically significant differences between control and KL-treated groups are indicated as follows: * *p* < 0.05; ** *p* < 0.01.

**Table 4:** Binding affinity, conventional hydrogen bonds, and the closest interacting residues of glyphosate with 3 Glu form of osteocalcin (3 Glu-OCN) (4MZZ) and decarboxylated osteocalcin (8I75).

Entry	Affinity (kcal/mol)	RMSD (Lower–Upper)	Interacting Residues	Closest Interacting Residue (Distance, Å)
4mzz	−3.5	0.0–10.49	**Attractive Charge:** GLU40 **Conventional H-Bond:** GLU40, ARG43, ARG43, ILE36, ILe36 **Carbon H-Bond:** GLU40	Glu40:HN (1.966)
8i75	−4.2	0.0–10.9	**Attractive Charge:** GLU24, ASP30, ASP34, ASP30, ASP34 **Conventional H-Bond:** ARG20, ASP30, ASP34, ASP34, ASP30, ASP34 **Carbon H-Bond:** GLU24	ASP30:OD2 (2.401)

## Data Availability

The data that support the findings of this study are available from the corresponding author upon reasonable request.
